# Improved Dose Response of *N*-(hydroxymethyl)acrylamide Gel Dosimeter with Calcium Chloride for Radiotherapy

**DOI:** 10.3390/gels8020078

**Published:** 2022-01-26

**Authors:** Khalid A. Rabaeh, Rawan E. Al-Tarawneh, Molham M. Eyadeh, Issra’ M. E. Hammoudeh, Moneeb T. M. Shatnawi

**Affiliations:** 1 Medical Imaging Department, Faculty of Applied Medical Sciences, The Hashemite University, Zarqa 13133, Jordan; 2 Physics Department, School of Science, The University of Jordan, Amman 11942, Jordan; rawantarawneh56@gmail.com (R.E.A.-T.); moneeb.shatnawi@ju.edu.jo (M.T.M.S.); 3 Physics Department, Faculty of Science, Yarmouk University, Irbid 21163, Jordan; molhem.e@yu.edu.jo; 4 School of Basic Sciences and Humanities, German Jordanian University, Amman 11180, Jordan; issrahammoudeh@outlook.com

**Keywords:** polymer gel, *N*-(hydroxymethyl)acrylamide, calcium chloride, nuclear magnetic resonance, dosimetry

## Abstract

The impact of calcium chloride (CaCl_2_) on the performance of *N*-(hydroxymethyl)acrylamide (NHMA) polymer gel dosimeter is studied in this article. The dosimeter was exposed to doses of up to 10 Gy with radiation beam-energy of 10 MV and dose-rates of 300 cGy/min. The relaxation rate (R_2_) parameter was utilized to explore the performance of irradiated NHMAGAT gels. The dose response in terms of R_2_ increased from 0.29 to 0.63 Gy^−1^·s^−1^ with increasing calcium chloride concentration from 0 to 1000 mM. The results show no substantial impact of dose-rates as well as radiation energies on NHMAGAT samples. For the steadiness of irradiated NHMAGAT dosimeters, it was found that there is no apparent variation in R_2_ (less than ±3%; standard deviation) up to 3 days. The overall uncertainty of the gel dosimeter with calcium chloride is 4.96% (double standard deviation, 95% confidence level).

## 1. Introduction

New developments in radiotherapy have been concerned with the study of equivalent gel dosimeters for tissues containing active chemical sensors to measure the absorbed radiation dose [1,2,3,4,5,6]. Gel dosimeters such as polymer gels have a range of properties like biological tissues, and are suitable alternatives compared with conventional dosimeters due to their ability in resolving three-dimensional (3D) dose distributions [7,8,9,10,11].

Polymer gels have an electron density that is like tissue-equivalent material [12,13,14]. It contains monomers that polymerize during ionizing radiation [15,16]. The first recipe was fabricated from N, N’methyelene-bis-acrylamide and acrylamide [17,18].

The complex radiation dose response recorded in polymer gels (i.e., degree of polymerization) can be explored in 3D utilizing various modalities such as MRI and optical techniques [18,19,20]. The interaction between monomers in the gel after irradiation leads to drops in the mobility of H_2_O molecules and decreases in the relaxation time (T_2_) values in the MRI technique [21,22,23,24]. These alterations of the transverse relaxation rate (R_2_ = 1/T_2_) can be measured via NMR relaxometry [25,26].

Different types of monomers were incorporated in the preparation of various types of polymer gels, such as N-isopropylacrylamide [27], methacrylic-acid [28,29], N-hydroxyethylacrylate [30], N-vinylpyrrolidone [31,32,33], Itaconic-acid [34], 2-acrylamide-2-methylpropane-sulfonic-acid sodium salt [35], N-(3-methoxypropyl)-acrylamide [10,36], and *N*-(hydroxymethyl)-acrylamide [8,37,38,39].

Many works have reported the influence of different salts on the performance of gel dosimeters. The literature shows a significant effect of specific inorganic salts on the dose response of irradiated gels [38,39,40,41,42]. The polymerization of polymer gels has been dramatically increased and enhanced by the addition of inorganic salts that attract water molecules and other elements via electrostatic interactions in the gel, leading to an increase in the rate of polymerization [40]. Rabeah et al. (2017) [37] introduced the composition of a polymer gel that contains *N*-(hydroxymethyl)acrylamide monomer (NHMA). The evaluation of irradiating this polymer gel was carried out by NMR and spectrophotometry. The data show a good response to ionizing radiation.

In this work, the major objective is to increase the dose sensitivity for formulations of NHMA gel samples by adding an appropriate concentration of salt, calcium chloride (CaCl_2_). The influence of temperature during scanning, dose-rates, radiation energies and the stability of irradiated samples were also examined and reported.

## 2. Materials and Methods

### 2.1. Samples Preparation

NHMAGAT samples were fabricated in normal conditions as the previous gel of normoxic polymer gel [12]. This polymer gel has five main components: NHMA (8 wt%) and N, N-methylene-bis-acrylamide (3 wt%) as a co-monomers, gelatin-type A (4 wt%) as a gel matrix, tetrakis (hydroxymethyl) phosphonium chloride-THPC (20 mM) as an oxygen scavenger, and a wide concentration range of calcium chloride (0–1000 mM) as an additive. The chemicals were purchased from Sigma-Aldrich chemical company (St. Louis, MO, USA). NHMAGAT polymer gels were fabricated as follows: at room temperature, gelatin-type A was added to the deionized-water and stirred for 5 min, Then, the temperature of mixture was increased to 48 °C for one hour using a hot-plate magnetic stirrer. Then, the NHMA, BIS, and CaCl_2_ were added and stirred until a homogenous solution had been obtained. After that, THPC was added to the solution at 35 °C and the solution was stirred for about 2 min. The prepared solutions were poured into air-tight NMR glass tubes (10 mL) and kept in a fridge for 24 h before X-ray irradiation.

### 2.2. Irradiation

The prepared gels were placed in a cubic water tank (30 × 30 × 30 cm^3^) and exposed to various absorbed doses at standard parameters of (5 cm depth, 100 source to surface distance and 20 × 20 cm^2^ field size) using 10 MV Linac X-ray beam (Elekta, Laurent Leksell, Stockholm, Sweden) with a 300 cGy/min dose-rate. The impact of dose-rates and radiation energies were examined by irradiating the gel dosimeters at a fixed beam energy of 10 MV with 150 and 600 cGy/min and at a fixed dose-rates of 300 cGy/min with 6 and 15 MV. Three gels were exposed at certain absorbed doses, and the average values are shown in the Results and Discussion section.

### 2.3. Nuclear Magnetic Resonance (NMR) Measurements

The prepared samples were placed into a water-bath system (Julabo, Seelbach, Germany), which was connected to an NMR relaximeter to control the scanning temperature during NMR measurement from 10 to 30 °C. The NMR samples were read out using the 0.5 T NMR technique (Bruker, Bremen, Germany) under a fixed scanning temperature of 20 °C. The relaxation rate (R_2_) values of the measured gels were calculated by a standard Multi-Spin-Echo sequence (Carr Purcell Meiboom Gill (CPMG)) at 0.4 ms echo-time spacing and 2000 echoes. Three gels were read out at certain absorbed doses, and the average values as well as standard deviations are included in the Results and Discussion section.

## 3. Results and Discussion

### 3.1. Impact of Calcium Chloride (CaCl_2_) Concentration

**Figure 1:**
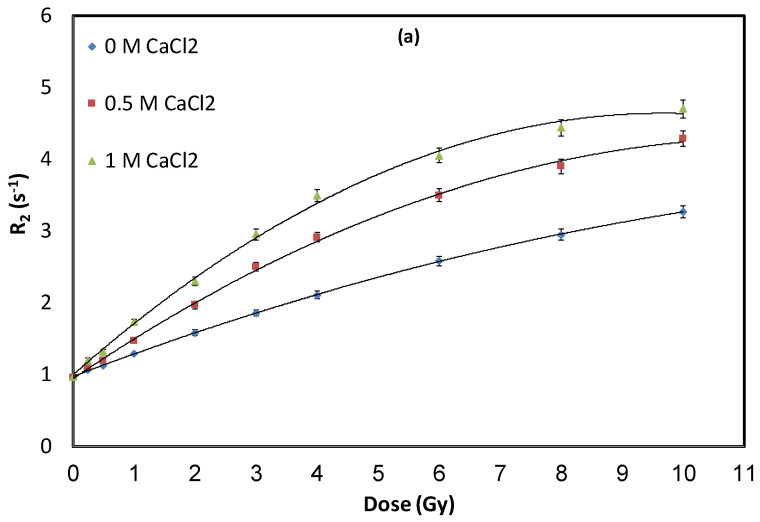

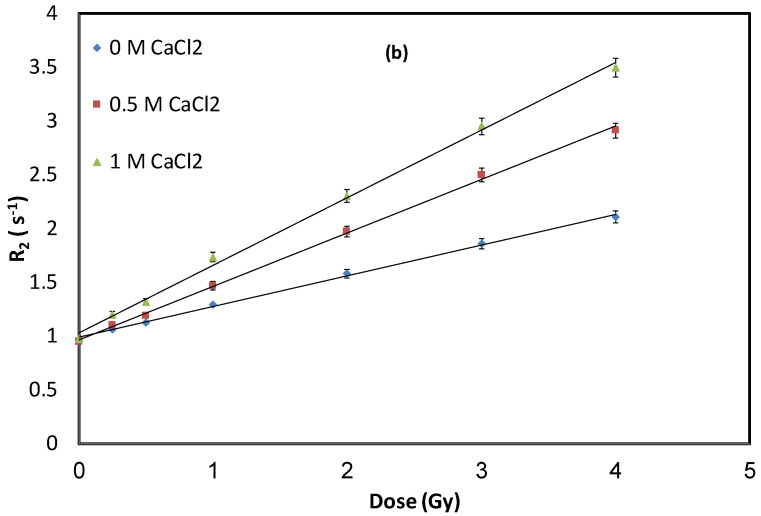
R_2_ of gels with various concentrations of CaCl_2_ when irradiated at doses up to (**a**) 0–10 Gy, and (**b**) 0−4 Gy. Error bars are the 2σ of R_2_ values.

**Figure 2:**
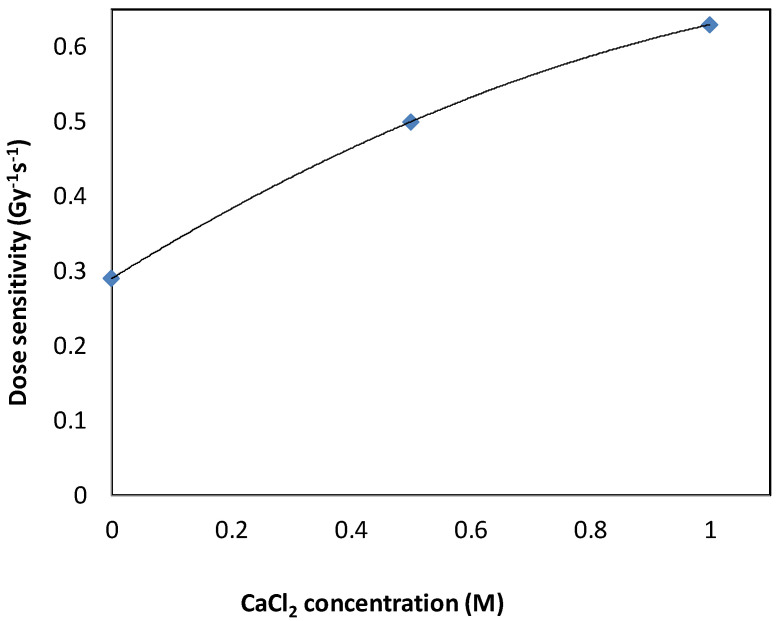
Dose sensitivity value for different CaCl_2_ concentrations.

Different compositions of NHMAGAT gel dosimeters, with different concentrations of CaCl_2_, were prepared to evaluate the impact of salt on the dose response and dose sensitivity of NHMAGAT dosimeters. A set of three samples from different gel batches were used for each formulation code in all absorbed dose points. Figure 1 shows the relationship between the relaxation rate and the dose amount, which proves that CaCl_2_ did not increase the background value of the unirradiated NHMAGAT dosimeter. Additionally, these curves of NHMAGAT samples show a remarkable increase in R_2_ with an absorbed dose up to 10 Gy because of increasing the polymerization of the NHMAGAT gel. When the CaCl_2_ concentration changes from zero to 1000 mM, the relaxation rate increases proportionally. This can be clarified by the hypothesis presented by Hayashi, which states that inorganic salts such as CaCl_2_ increase the temperature of gels during irradiation, and as a result, the exothermic polymerization rate increases, which improves the dose sensitivity [40]. To override the effect of salt on the reduction in the melting point of the prepared gel [43], a double amount of the antioxidant (20 mM) was used. Additionally, in Figure 1, the intercept is related directly to the gel matrix [22]. In contrast, the sensitivity obtained from the slope of linear fit was found from the slope of the linear part in the R_2_ dose graph, which determines the dose resolution [44,45]. The R_2_ curves are approximated by linear-fit in a dose-range of 0–4 Gy (see Figure 1b). Beyond 4 Gy, the response changes towards the saturation region due to the increase in the consumption rate of co-monomers after adding salt (Figure 1a). The sensitivity increases strongly when the CaCl_2_ concentration is increased from 0 to 1000 mM (see Figure 2 and Table 1).

### 3.2. Stability of NHMAGA Dosimeters

**Figure 3:**
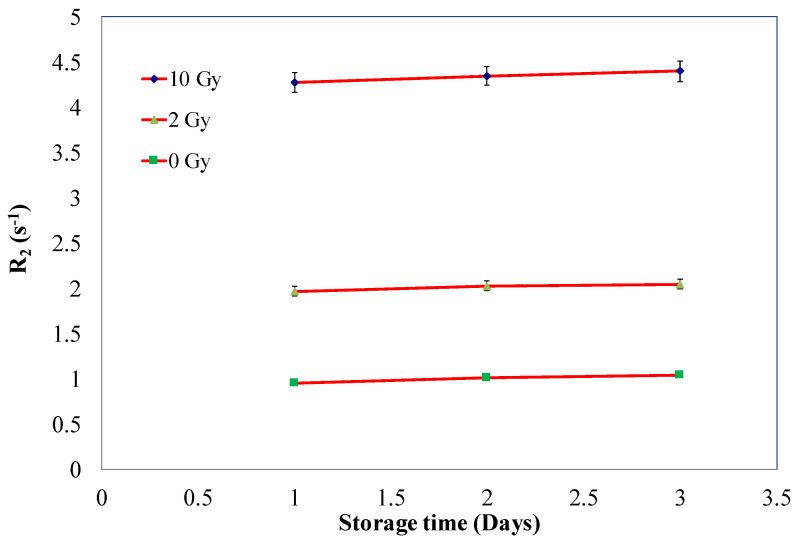
R_2_ of gel with 500 mM CaCl_2_ irradiated at different doses as a function of storage time. Error bars are the 2σ of R_2_ values.

The NHMAGAT (2) gel dosimeter was irradiated at three different doses and kept in a fridge for three days to study the effect of post-irradiation stability. Three gels for each dose were used, and the average values are presented. The samples were read out daily for 3 days, which is quite suitable for routine dose calibration. The results are shown in Figure 3, from which it is evident that there is no significant change in R_2_ after up to 3 days.

### 3.3. Influence of Dose-Rates

**Figure 4:**
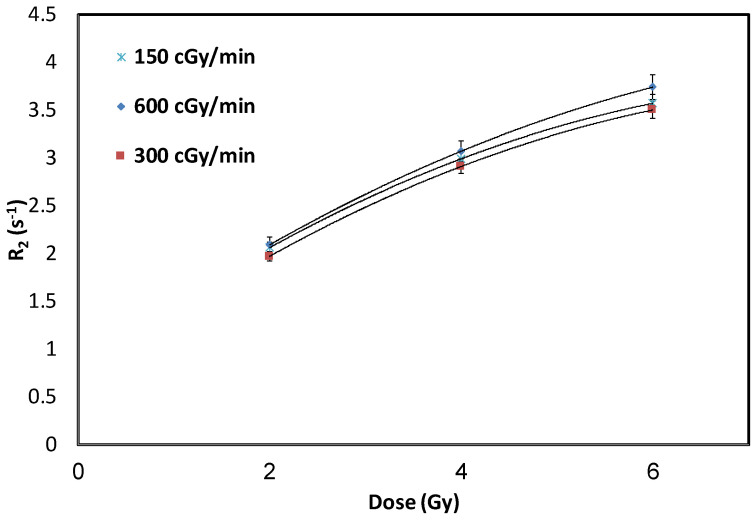
R_2_ of gel with 500 mM CaCl_2_ for various dose-rates under 10 MV radiation energy. Error bars are the 2σ of R_2_ values.

NHMAGAT with 500 mM CaCl_2_ was used to investigate the influence of dose-rates, i.e., 150, 300 and 600 cGy/min, with a constant radiation energy of 10 MV. These gel samples were exposed to various doses of 2, 4, and 6 Gy. To report the standard deviations, a set of three gels were exposed to each selected dose. Figure 4 shows no significant effect of dose-rates on this polymer gel dosimeter.

### 3.4. Effect of Radiation-Energies

**Figure 5:**
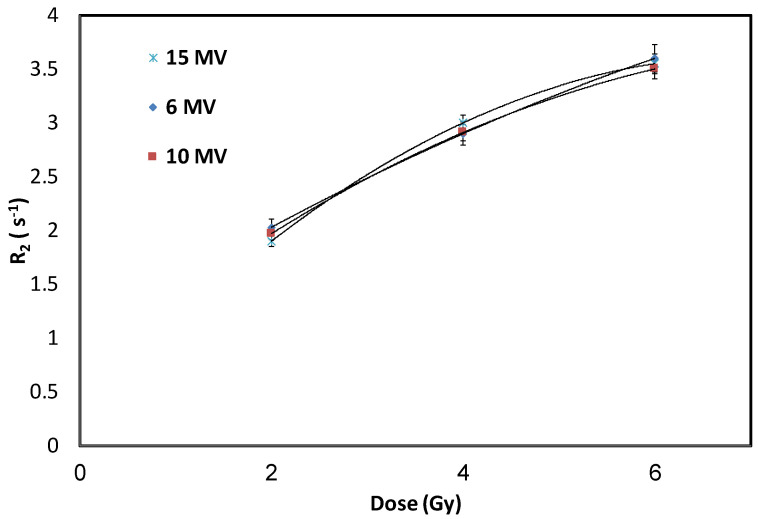
R_2_ of gel with 500 mM CaCl_2_ for various radiation energies. Error bars are the 2σ of R_2_ values.

The impact of radiation energies on the performance of NHMAGAT with 500 mM CaCl_2_ was studied by exposing the gel to three different values of radiation energy at dose-rates of 300 cGy/min. These gels were exposed to different absorbed doses. The results in Figure 5 show that there is little influence of radiation dose on this polymer gel.

### 3.5. Effect of Scanning Temperature

**Figure 6:**
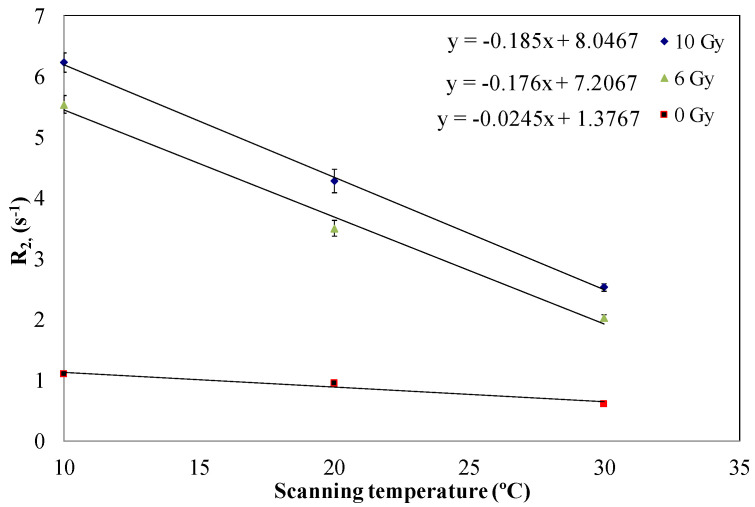
Relaxation rate of unirradiated and irradiated gels with 500 mM CaCl_2_ exposed to 6 and 10 Gy as a function of scanning temperature. Error bars are the 2σ of R_2_ values.

Samples of formulation code NHMAGAT (2) were scanned at different temperatures. Figure 6 illustrates that the response increases with the cooling of the samples during the NMR scanning. This significant change in NMR readout is due to the alteration in the magnetization values between the protons of the semi-solid and aqueous phase that regulate the relaxation time of H_2_O in the gel, which is enhanced with declining scanning temperature [46,47,48].

### 3.6. Uncertainty Analysis

The overall uncertainty budget for the dosimeter was calculated based on different parameters of uncertainty. These parameters are the calibration of the radiotherapy unit with the ionization chamber (1.2%), batch uniformity (1.4%), reproducibility of measurements (1.1%), MNR sensitivity variation (0.3%), calibration curve fit (1.2%), and temporal stability (0.2%) [2,49].

The calculated total dose of uncertainty is defined as the combined uncertainty multiplied by two for a confidence level of 95% [49]. The square root of the sum of all uncertainty components is equal to the combined uncertainty [49]. Therefore, the combined uncertainty is 2.48% and the calculated overall uncertainty for R_2_ measurements is 4.96%. These values are considered sufficient for the estimation of dose distributions in radiotherapy.

## 4. Conclusions

An improved composition of polymer gel dosimeter has been introduced as a normoxic polymer gel *N*-(hydroxymethyl)acrylamide (NHMAGAT) for radiotherapy by the addition of CaCl_2_ inorganic salt. The results of the NHMAGAT gel dosimeter in this study show that the response to the dose increases strongly with increasing CaCl_2_ concentration from 0 to 1 M. The unirradiated and irradiated developed gel samples were almost stable after up to 3 days. No appreciable effect was observed on the performance of irradiated NHMAGAT gel dosimeter when changing the dose-rates or radiation energies. The response increases strongly upon cooling the gel during NMR measurement. Overall, the data suggest strongly that CalCl_2_ acts as an excellent sensitizer to the polymerization of irradiated dosimeters.

## Figures and Tables

**Table 1:** Linear equations and sensitivities of curves in Figure 1b.

Linear Equations	Sensitivity (Gy^−1^·s^−1^)	Recipe
R_2_ = 0.29D + 0.99	0.29	NHMAGAT (1)
R_2_ = 0.50D + 0.96	0.50	NHMAGAT (2)
R_2_ = 0.63D + 1.03	0.63	NHMAGAT (3)

## Data Availability

The study did not report any data.

## References

[1] Rabaeh K.A., Basfar A.A., Moussa A.A., Msalam R.I. (2013). Novel radio-chromic solution dosimeter for radiotherapy treatment planning. Phys. Med..

[2] Soliman Y.S. (2014). Gamma-radiation induced synthesis of silver nanoparticles in gelatin and its application for radiotherapy dose measurements. Radiat. Phys. Chem..

[3] Eyadeh M.M., Rabaeh K.A., Hailat T.F., Aldweri F.M. (2018). Evaluation of ferrous Methylthymol blue gelatin gel dosimeters using nuclear magnetic resonance and optical techniques. Radiat. Meas..

[4] Soliman Y.S., Beshir W.B., Abdelgawad M.H., Brauer-Krisch E., Abdel-Fattah A.A. (2019). Pergascript orange-based polymeric solution as a dosimeter for radiotherapy dosimetric validation. Phys. Med..

[5] Watanabe Y., Mizukami S., Eguchi K., Maeyama T., Hayashi S.-I., Muraishi H. (2009). Dose distribution verification in high-dose-rate brachytherapy using a highly sensitive normoxic N-vinylpyrrolidone polymer-gel dosimeter. Phys. Med..

[6] Rabaeh K.A., Eyadeh M.M., Hailat T.F., Aldweri F.M., Alheet S.M., Eid R.M. (2018). Characterization of ferrous-methylthymol blue-polyvinyl alcohol gel dosimeters using nuclear magnetic resonance and optical techniques. Radiat. Phys. Chem..

[7] Adliene D., Jakstas K., Vaiciunaite N. (2014). Application of optical methods for dose evaluation in normoxic polyacrylamide gels irradiated at two different geometries. Nucl. Instrum. Methods Phys. A.

[8] Basfar A.A., Moftah B., Rabaeh K.A., Almousa A. (2015). Novel composition of polymer-gel dosimeters based on N-(Hydro-xymethyl) acrylamide for radiation therapy. Radiat. Phys. Chem..

[9] Maeyama T., Ishida Y., Kudo Y., Fukasaku K., Ishikawa K.L., Fukunishi N. (2018). Polymer-gel dosimeter with AQUAJOINT^®^ as hydrogel matrix. Radiat. Phys. Chem..

[10] Awad S.I., Moftah B., Basfer A., Almousa A.A., Al Kafi M.A., Eyadeh M.M., Rabaeh K.A. (2019). 3-D Quality Assurance in CyberKnife Radiotherapy Using a Novel N-(3-methoxypropyl) Acrylamide Polymer-gel Dosimeter and Optical CT. Radiat. Phys. Chem..

[11] Rabaeh K.A., Al-Ajaleen M.S., Abuzayed M.H., Aldweri F.M., Eyadeh M.M. (2019). High dose sensitivity of N-(isobutoxymethyl)acrylamide polymer-gel dosimeters with improved monomer solubility using acetone co-solvent. Nucl. Instrum. Methods Phys. Res. Sect. B.

[12] De Deene Y., Vergote K., Claeys C., De Wagter C. (2006). The fundamental radiation properties of normoxic polymer-gel dosimeters: A comparison between a methacrylic acid-based gel and acrylamide-based gels. Phys. Med. Biol..

[13] Baldock C., De Deene Y., Doran S., Ibbott G., Jirasek A., Lepage M., McAuley K.B., Oldham M., Schreiner L.J. (2010). Polymer-gel dosimetry. Phys. Med. Biol..

[14] Tachibana H., Watanabe Y., Mizukami S., Maeyama T., Terazaki T., Uehara R., Akimoto T. (2020). End-to-end delivery quality assurance of computed tomography–based high-dose-rate brachytherapy using a gel dosimeter. Brachytherapy.

[15] Maryanski M.J., Gore J.C., Kennan R.P., Schulz R.J. (1993). NMR relaxation enhancement in gels polymerized and cross-linked by ionizing radiation: A new approach to 3D dosimetry by MRI. Magn. Reson. Imaging.

[16] Mizukami S., Watanabe Y., Mizoguchi T., Gomi T., Hara H., Takei H., Fukunishi N., Ishikawa K.L., Fukuda S., Maeyama T. (2021). Whole Three-Dimensional Dosimetry of Carbon Ion Beams with an MRI-Based Nanocomposite Fricke Gel Dosimeter Using Rapid T1 Mapping Method. Gels.

[17] De Deene Y. (2002). Gel dosimetry for the dose verification of Intensity Modulated Radiotherapy Treatments. Z. Med. Phys..

[18] Gore J.C., Ranade M., Maryanski M.J., Schulz R.J. (1996). Radiation dose distributions in three dimensions from tomographic optical density scanning of polymer-gels: I. Development of an optical scanner. Phys. Med. Biol..

[19] Oldham M., Siewerdsen J.H., Shetty A., Jaffray D.A. (2001). High resolution gel-dosimetry by optical-CT and MR scanning. Med. Phys..

[20] Rabaeh K.A., Saion E., Omer M., Shahrim I., Alrahman A.A., Hussain M. (2008). Enhancements in 3D Dosimetry Measurement using Polymer-gel and MRI. Radiat. Meas..

[21] Ibbott G.S., Maryanski M.J., Eastman P., Holcomb S.D., Zhang Y., Avison R.G., Sanders M., Gore J.C. (1997). Three-dimensional visualization and measurement of conformal dose distributions using magnetic resonance imaging of BANG polymer-gel dosimeters. Int. J. Radiat. Oncol. Biol. Phys..

[22] De Deene Y., De Wagter C., Van Duyse B., Derycke S., Mersseman B., De Gersem W., Voet T., Achten E., De Neve W. (2000). Validation of MR-based polymer-gel dosimetry as a preclinical three-dimensional verification tool in conformal radiotherapy. Magn. Reson. Med..

[23] Pappas E., Maris T. (2020). Polymer-gel 3D dosimetry in radiotherapy. Z. Med. Phys..

[24] Rabaeh K.A., Issra’ME H., Oglat A.A., Eyadeh M.M., Ala’J A.Q., Aldweri F.M., Awad S.I. (2021). Polymer-gel containing N, N′-methylene-bis-acrylamide (BIS) as a single monomer for radiotherapy dosimetry. Radiat. Phys. Chem..

[25] El-Khayatt A.M. (2017). Water equivalence of some 3D dosimeters: A theoretical study based on the effective atomic number and effective fast neutron removal cross section. Nucl. Sci. Tech..

[26] De Deene Y. (2020). Gel-based Radiation Dosimetry Using Quantitative MRI. NMR MRI Gels.

[27] Koeva V., Olding T., Jirasek A., Schreiner L., McAuley K. (2009). Preliminary investigation of the NMR, optical and x-ray CT dose–response of polymer-gel dosimeters incorporating co solvents to improve dose sensitivity. Phys. Med. Biol..

[28] Trapp J.V., Partridge M., Hansen V.N., Childs P., Bedford J., Warrington A.P., Leach M.O., Webb S. (2004). The use of gel dosimetry for verification of electron and photon treatment plants in carcinoma of the scalp. Phys. Med. Biol..

[29] Gopishankar N., Vivekanandhan S., Kale S.S., Rath G.K., Senthilkumaran S., Thulkar S., Subramani V., Laviraj M.A., Bisht R.K., Mahapatra A.K. (2012). MAGAT gel and EBT2 film-based dosimetry for evaluating source plugging-based treatment plan in Gamma Knife stereotactic radiosurgery. J. Appl. Clin. Med. Phys..

[30] Rabaeh K.A., Saion E., Omer M.A.A., Rahman A.A., Hussain M.Y., Iskandar S., Ali N.M. (2008). Rate of Elapsed Polymerization of Hydroxyethylacrylate Gel Induced by Gamma Radiation. Nucl. Sci. Tech..

[31] Pappas E., Maris T., Angelopoulos A., Paparigopoulou M., Sakelliou L., Sandilos P., Voyiatzi S., Vlachos L. (1999). A new polymer-gel for magnetic resonance imaging (MRI) radiation dosimetry. Phys. Med. Biol..

[32] Kozicki M., Maras P., Rybka K., Biegański T. (2009). VIPARnd—GeVero^®^ tool in planning of TPS scheduled brain tumour radiotherapy. J. Phys. Conf. Ser..

[33] Kozicki M., Berg A., Maras P., Jaszczak M., Dudek M. (2020). Clinical radiotherapy application of N-vinylpyrrolidone-containing 3D polymer-gel dosimeters with remote external MR-reading. Phys. Med..

[34] Mattea F., Chacón D., Vedelago J., Valente M., Strumia M.C. (2015). Polymer-gel dosimeter based on itaconic acid. Appl. Radiat. Isot..

[35] Farhood B., Abtahi S.M., Geraily G., Ghorbani M., Mahdavi S.R., Zahmatkesh M.H. (2018). Dosimetric characteristics of PASSAG as a new polymer-gel dosimeter with negligible toxicity. Radiat. Phys. Chem..

[36] Moftah B., Basfar A., Almousa A., Al Kafi A., Rabaeh K. (2020). Novel 3D polymer-gel dosimeters based on N-(3-Methoxypropyl)acrylamide (NMPAGAT) for quality assurance in radiation oncology. Radiat. Meas..

[37] Rabaeh K.A., Basfar A.A., Almousa A.A., Devic S., Moftah B. (2017). New normoxic *N*-(hydroxymethyl)acrylamide based polymer-gel for 3D dosimetry in radiation therapy. Phys. Med..

[38] Rabaeh K.A., Salman N.M.B., Aldweri F.M., Saleh H.H., Eyadeh M.M., Awad S.I., Oglat A.A. (2021). Substantial influence of magnesium chloride inorganic salt (MgCl2) on the polymer dosimeter containing *N*-(hydroxymethyl)acrylamide for radiation therapy. Results Phys..

[39] Rabaeh K.A., Issra’ME H., Eyadeh M.M., Aldweri F.M., Awad S.I., Oglat A.A., Shatnawi M.T. (2021). Improved performance of *N*-(hydroxymethyl)acrylamide gel dosimeter using potassium chloride for radiotherapy. Radiat. Meas..

[40] Hayashi S.I., Fujiwara F., Usui S., Tominaga T. (2012). Effect of inorganic salt on the dose sensitivity of polymer-gel dosimeter. Radiat. Phys. Chem..

[41] Hayashi S.I., Kawamura H., Usui S., Tominaga T. (2013). Comparison of the influence of inorganic salts on the NMR dose sensitivity of polyacrylamide-based gel dosimeter. J. Phys. Conf. Ser..

[42] Hayashi S.I., Kawamura H., Usui S., Tominaga T. (2018). Influence of magnesium chloride on the dose–response of polyacrylamide-type gel dosimeters. Radiol. Phys. Technol..

[43] Chacón D., Strumia M., Valente M., Mattea F. (2018). Effect of inorganic salts and matrix crosslinking on the dose response of polymer-gel dosimeters based on acrylamide. Radiat. Meas..

[44] De Deene Y., van de Walle R., Achten E., de Wagter C. (1998). Mathematical analysis and experimental investigation of noise in quantitative magnetic resonance imaging applied in polymer-gel dosimetry. Signal Process..

[45] Baldock C., Lepage M., Bäck S.Å.J., Murry P.J., Jayasekera P.M., Porter D., Kron T. (2001). Dose resolution in radiotherapy polymer-gel dosimetry: Effect of echo spacing in MRI pulse sequence. Phys. Med. Biol..

[46] Gochberg D.F., Fong P.M., Gore J.C. (2001). Studies of magnetization transfer and relaxation in irradiated polymer-gels-interpretation of MRI-based dosimetry. Phys. Med. Biol..

[47] Lepage M., Jayasakera P.M., Bäck S.Å.J., Baldock C. (2001). Dose resolution optimization of polymer-gel dosimeters using different monomers. Phys. Med. Biol..

[48] Eyadeh M.M., Smadi S.A., Rabaeh K.A., Oglat A.A., Diamond K.R. (2021). Effect of lithium chloride inorganic salt on the performance of *N*-(hydroxymethyl)acrylamide polymer-gel dosimeter in radiation therapy. J. Radioanal. Nucl. Chem..

[49] ISO/ASTM 51707 (2015). Guide for Estimating Uncertainties in Dosimetry for Radiation Processing. Annual Book of ASTM Standards.

